# 

**Published:** 1905-07-15

**Authors:** 


### ANNOUNCEMENTS.

New Deans.—Dr. Charles A. Merrill has resigned as dean of the Birmingham Dental College and Dr. B. G. Copeland has been elected to succeed him. Dr. W. B. Fulton remains as secretary.—Dr. W. P. Dickerson has resigned as dean of the Dental Department of the University of Minnesota, and Dr. Alfred Owre has been appointed to succeed him.—Dr. G. S. Junkerman of the Cincinnati College of Dental Surgery has had conferred upon him the Honorary Degree of Master of Arts by the Ohio University of Athens, Ohio.

A NEW DENTAL JOURNAL.—The Cogswell Dental Supply Company, of Cleveland, Ohio, announces a new dental journal to be issued in September, and is inviting suggestions for a name. Offering a prize of $25.00 for the best name suggested. It is promised that the journal will be unique. Every article published in its pages will be paid for. If they pay enough, the profession will have a new source of income.

—✦—

THE MISSOURI STATE ASSOCIATION.—This society met in St. Louis, May 24th to 26th, and elected Dr. W. M. Carter, of Sedalia, President; Dr. Sam'l T. Bassett, Century Building, St. Louis, Secretary. The next meeting will be held next May in Springfield.

—✦—

ILLINOIS STATE DENTAL SOCIETY.—The largest meeting this society ever held was held at Moline, May 9th to 11th. Between seven and eight hundred members attended. The following officers were elected for next year: S. F. Duncan, of Joliet, President; Elgin MaWinney, of Chicago, Secretary. The next meeting will be held at Springfield, May 8th to 11th, 1906. All the local and district societies of the State are now affiliated with the State society, making a membership of over one thousand.

—✦—

NEW JERSEY STATE SOCIETY.—This society will hold its annual meeting at Asbury Park, July 19th, 20th and 21st. The clinical committee invites any practitioner to bring any difficult case for diagnosis or treatment, July 21st. Advice will be given or treatment made as may be desired by specialists in various lines of treatment. If case is reported to the chairman before the meeting, he will have some competent practitioner to advise or operate at the meeting.

Address Dr. J. E. Halsey,
Swedesboro, N. J.

